# ‘Forgetting’ or ‘Precipitation’: Literary inquisition in Qing Dynasty and modern enterprise risk preference

**DOI:** 10.1371/journal.pone.0300639

**Published:** 2024-03-22

**Authors:** Weizhou Wang, Weihua Yu, Jinfei Niu

**Affiliations:** 1 Jinhe Center for Economic Research, Xi’an Jiaotong University, Xi’an, China; 2 School of Finance and Data Science, Xi’an Eurasia University, Xi’an, China; Bahir Dar University, ETHIOPIA

## Abstract

This paper takes the risk preference of modern listed companies as the research object, uses the financial data of Chinese listed companies combined with the literary inquisition file in Qing Dynasty to conduct an empirical study, and examines the influence of literary inquisition on the risk preference of modern corporate CEOs in Qing Dynasty. The study found that the literary inquisition incident in Qing Dynasty significantly affected and reduced the risk preference of modern enterprises. The competitive hypothesis of the influence of Confucian culture and China City Commercial Credit Environment Index (CEI) on CEOs’ risk preference is excluded. In addition, through the study of heterogeneity, this paper also verifies that the influence of literary inquisition is more significant in areas with a higher degree of marketization, indicating that the influence of informal institutions depends on the establishment of formal institutions. Finally, in the mechanism study, this paper points out that the rulers’ suppression of ideas will change long-term social capital and lead to the decrease of general trust in society, which will make the enterprise managers born in the region tend to be conservative in their risk preference.

## 1 Introduction

There was little religious persecution in China, but the persecution of literature sometimes reached the level of the Inquisition, the worst of which occurred under the Manchus (Qing Dynasty), and have a long-term impact on human capital [1]. This informal system has a strong influence on the behavior of various economic actors in the market, including enterprises [2]. This paper examines how literary inquisition in Qing Dynasty affects the risk preference of modern enterprises. Enterprise risk preference refers to the willingness of enterprises to take risks in the process of pursuing high profits [3]. This is an important characteristic of a successful business. However, to the best of our knowledge, there are few empirical studies demonstrating the causal role of literary inquisitions in shaping corporate risk-taking.

There are significant differences in risk appetite in modern market behavior. As the main decision-maker of enterprises, entrepreneurs’ risk appetite directly affects the intensity of R&D investment and then affects the performance of enterprises [4]. The research in institutional economics finds that in the process of marketization and industrialization, informal institutions are a very complex and actually existing rule network, which is the result of historical accumulation and cultural evolution [5–8]. In recent years, the analysis of economic problems through informal institutions such as culture has become a deep-seated perspective. The influence of this aspect does shape the difference in enterprise risk preference [9, 10] and then affect economic development, especially the endogenous influence of measured cultural tradition and value concepts on standard economic growth [11].

Diana and David [12] expounded on the significant impact of informal institutions related to social capital on human behavior and welfare in the form of a theoretical review and dialectically described their impact on economic development and the marketization trend. As for how to influence the decision-making behavior and degree of enterprises, the study clearly found that the greater the influence of Confucian culture on a company, the lower the level of risk-taking [13]. Liang Xiong Huang and Ming hui Ma [3] discussed how the patriarchal culture in Chinese history reduces the risk-taking spirit of Chinese enterprises. It is verified that in places with strong family culture, senior executives (presidents and CEOs) will make enterprises more conservative, and corporate financing and investment behaviors in regions with higher risk aversion will be more conservative, and even the geographical relationship between CEOs and directors will significantly increase the risk of corporate financial decision-making [14]. In terms of how to influence corporate governance, more people think that it is related to the values and ways of thinking of the management. A study of high-tech enterprises finds that the overconfidence of senior executives will promote the investment and output of innovation projects [15], and the cultural differences of CEOs affect the quality of internal control [16]. The richer the career experience of CEOs, the higher the level of corporate risk-taking, and their rich career experience, as a self-restraint mechanism, presents a substitute relationship with external governance to some extent [17]. A CEO’s quest for profit always comes with risk. John et al. (2008) established a significant positive correlation between risk taking and the growth rate of enterprise assets and sales revenue [18]. There are two factors that affect enterprise risk taking: internal and external [19, 20]. The former includes: company scale, business performance, industry category, internal governance structure, individual differences in managers, such as age, gender, and experience. The latter include: macroeconomics, government policies and institutions, uncertainty brought about by environmental change.

During the Prosperous Qing Dynasty (1660–1794), graduates of the Imperial examination system faced systematic and large-scale persecution for the first time in Chinese history [21], These persecutions were called literary trials. Because the literary inquisition specifically targeted exam graduates and punished them for their writing. The main problem facing the Qing emperors was how to "dominate the cultured and highly sophisticated Chinese elite" [22]. In order to do this, the Qing rulers often investigated and punished the literati for what they wrote [23]. Only in terms of the number of literary inquisitions, the Qing Dynasty literary inquisitions should be about 160–170, more than the total number of literary inquisitions in other dynasties in history, stretching for more than 130 years.

The purpose of literary inquisition was to create an atmosphere of fear among the scholar-officials. In this respect, they are more like Stalin-era trials than persecutions in early modern Europe, which often had a religious character [24, 25]. Academics are investigated for arbitrary reasons, often mere suspicion. "The rulers are the only interpreters in these cases, and some of the allegations are based on suspicion" [26, 27].

The influence of literary inquisition on contemporary economy is concentrated on social capital, and the theoretical mechanisms verified by existing studies are mainly human capital and trust degree. The most immediate impact is that the short-term effect of the literary inquisition persecution will lead to a decrease in the number of people taking the imperial examination, and the long-term effect is to reduce the provision of basic education in the early 20th century, and have a lasting impact on human capital accumulation [28], even in modern China. The victims of literary inquisitions are still discussed in their hometowns [29]. Studies have shown that the ‘upper tail’ knowledge of educated elites played a key role in disseminating the Industrial Revolution [30], and these studies have established that the effects of shocks can persist for decades or even centuries [31]. Acemoglu and Robinson [32] observe that national policies and behaviors influence the nature and extent of social capital, i.e. the attitudes, beliefs, norms, and perceptions that support cooperation. Gong Zizhen (1792–1841, Thinker, poet, litterateur and pioneer of reformism in Qing Dynasty) believes that the fear of persecution leads intellectuals to withdraw from society and withdraw from public life. Further research shows that the Qing Dynasty literary inquisition is negatively correlated with today’s general trust, and verifies that the suppression of literary inquisition permanently reduces social capital from several dimensions such as basic education, political participation and democratic attitudes, and less social trust reduces risk-taking. Based on this, this paper hypothesizes that the region where literary inquisition occur will reduce the risk appetite of CEOs.

As for the study of literary inquisition in Qing Dynasty, this paper makes an in-depth study according to the place of the event, the people involved and the scope of influence, and matches the division of administrative areas in Qing Dynasty with modern cities. Some events are screened out, and the occurrence of such events may have the opposite effect. For example, in the case of Chen Anzhao’s book writing in Changsha, Hunan Province in 1757, the emperor did not pursue his crime, which was obviously the opposite of the impact of other literary inquisition events. In addition, this paper divides the place of literary inquisition into different levels according to the county level and the city level, so as to facilitate the subsequent matching of different levels.

On the other hand, the volatility of stock returns of Chinese listed companies is used as a proxy variable for risk appetite [18, 33–35], constructed panel data by matching the birthplace of CEOs of these listed companies with the prefecture-level city data where literary inquisitor occurred, and used two different models to empiratically test the relationship between literary inquisitor and the risk preference of CEOs of enterprises. The empirical results confirm the basic hypothesis that literary inquisitor significantly reduces CEO risk appetite and show robust results in different regression models.

In order to ensure the reliability of the results, this paper also did a series of robustness tests. First, different methods are used here to calculate a firm’s risk appetite, using the range method and using different years of data, and the empirical results remain robust. Secondly, explanatory variables were matched using county-level data, the empirical results are still significant. It should be noted that the reason why the basic regression does not use county-level data is because some county-level literary inquisitions have not been born CEOs, which may lead to selective bias, but as a robustness test, it meets the requirements.

In order to further examine the interaction between formal institutions (marketization) and informal institutions (literary inquisition) [36]. This paper used the marketization index [37] to test on behalf of the formal system, and found that where the degree of marketization is stronger, literary inquisition will have a more significant impact on the risk preference of enterprises. This result confirmed that the role of informal system is based on the formal system. It also further confirmed the effectiveness of literary inquisition. At the same time, our heterogeneous study based on the nature of listed companies shows that the risk appetite of non-state-owned enterprises is more significantly affected by literary inquisition [38].

In order to improve the credibility, the competitive hypothesis is excluded. The literary inquisition was mainly a political oppression of scholars in the Qing Dynasty, which means that the literary inquisition explosion may also be in the concentrated areas of scholars, is it the literary inquisition or Confucian culture to reduce the risk appetite of enterprises [3]? We collected the information of all 25229 Jinshi in Qing Dynasty as proxy variables of Confucian culture for empirical research. The results show that both literary inquisition and Confucian culture can significantly reduce the risk appetite of enterprises, but they do not replace each other. The influence of literary inquisition is still significant, which further confirms the effectiveness of literary inquisition on the risk appetite of enterprises.

Finally, the paper examines the mechanism of literary inquisition. We used data from the 2015 Chinese Comprehensive Social Survey (CGSS2015) to test the level of social trust in the literary-prison area [39], and the results showed that the level of social trust in the literary-prison area was significantly lower than that in the non-literary-prison area. It is proved that the decrease of enterprise risk appetite is the result of the influence of regional social capital on literary inquisition [40], and this influence continues up to now.

This paper makes the following contributions to existing research. First, it’s an expansion of the literature on enterprise risk appetite. Existing studies have more influences on enterprise risk appetite as an independent variable, including company profit [41], R&D investment [4], company investment [42], and enterprise growth [43]. There are few studies on what factors affect enterprise risk preference, and a small number of studies focus on the influence of Confucian culture [13], manager heterogeneity [44], and patriarchal culture [3] on enterprise risk preference, while few studies involve the influence of major historical and cultural events. Secondly, previous studies mainly discussed the influence of literary inquisitions on cultural ecology, literary works, imperial power, and the balance of Taoism [29, 45, 46] and its impact on contemporary human capital and social capital [28]. This paper found that literary inquisitions affect enterprise risk preference through social trust and verified the communication mechanism of literary inquisition, which also enriched the research on literary inquisition. Thirdly, through the study of the competitive hypothesis of Confucian culture, it is found that the dual influence of Confucian culture and literary inquiry proves the pluralism of the influence of informal institutions. Heterogeneity research provides a certain reference for understanding the difference in risk appetite of different enterprises and different degrees of marketization and for studying differentiated governance.

The structure of this paper is as follows: The second part is the research design, describing the model setting, main variables, data sources, and descriptive statistics. The third part is the empirical results. The fourth part is the study of heterogeneity. The fifth part excludes the competitive hypothesis, while the sixth part discusses the mechanism and finally summarizes the thesis.

## 2 Research design

### 2.1 Samples selection

Based on the information provided in the ‘Archives of Literary Inquisition in Qing Dynasty (Updated Edition)’, this paper statistics the most concentrated literary inquisition cases during the period of the outbreak of literary inquisition in Qing Dynasty from 1647 (the 4th year of Shunzhi) to 1788 (the 53rd year of Qianlong) in a total of 141 years. The selected sample includes a total of 98 major recorded literary inquisition cases, including 4 in the Shunzhi Period. There were 5 cases in the Kangxi period, 5 cases in the Yongzheng period, and 84 cases in the Qianlong period, involving more than 130 county-level regions and tens of thousands of people involved.

The number of listed companies in the early stages is small, the company samples are not comprehensive, most of them are state-owned enterprises listed companies, and more importantly, the basic information of CEOs of enterprises is missing, so it is impossible to obtain effective samples. Therefore, in order to select a relatively continuous and stable sample, this paper selects the period from 2012 to 2019, obtains more than 2,000 listed companies with complete CEO information data, and the sample selection meets the basic requirements of random sampling.

### 2.2 Variable definition

#### 2.2.1 Core explanatory variable

The prefectural capitals of Qing Dynasty were matched with the prefectural cities and counties of modern times, and the literary inquisition involved more than 130 county-level regions and more than 40 county-level regions. Then, these data were matched with the birthplace of CEOs at the prefectural and municipal levels, and compiled into virtual variables of the occurrence of literary inquisition and the absence of literary inquisition, which constituted the core explanatory variable of the influence investigated in this paper (*LiteraryIB*).The native place of CEO was matched with the place of literary inquisition to form another explanatory variable (*LiteraryIN*), which was used as a substitute explanatory variable for robustness test. In addition, the county data were used for matching, and the county birthplace matching explanatory variables (*LiteraryICB*) and county birthplace matching explanatory variables (*LiteraryICN*) were obtained. Although the county data were accurate, the 61 locations involved in the county data were not matched, and were only used as alternative explanatory variables in the robustness test.

#### 2.2.2 Explained variable

Data related to the listed companies selected in the sample are used to measure the risk appetite. In this paper, the measurement method proposed by John et al. [18] is used to measure the risk appetite of enterprises by the volatility of earnings for three consecutive years, σ(*Adj*_*ROA*_*i*,*t*_). The specific calculation method can be found in the following formula:

$$Risk=\sqrt{\frac{1}{T-1}\sum_{t=1}^{T}{\left({Adj\_ROA}_{i,t}-\frac{1}{T}\sum_{t=1}^{T}{Adj\_ROA}_{i,t}\right)}^{2}|T\geq 3}$$

$$s.t.\;{Adj\_ROA}_{i,t}=\frac{{EBITDA}_{i,t}}{{ASSETS}_{i,t}}-\frac{1}{N_{t}}\sum_{k=1}^{N_{t}}\frac{{EBITDA}_{k,t}}{{ASSETS}_{k,t}}$$

Where *Adj*_*ROA*_*i*,*t*_ is the ratio of enterprise *i*’s earnings before interest, tax and depreciation (*EBITDA*) adjusted by the average value of the industry in year t to the total assets at the end of year (*ASSETS*).Based on the above variance calculation results, a relatively accurate value of risk preference of listed companies in three consecutive years is obtained as the core explained variable (*RiskAVar*).The range results were calculated using the above method to substitute the explained variable for the robustness test (*RiskARan*).

#### 2.2.3 Control variable

With reference to the existing literatures, the logarithm of urban population (*lnpop*), logarithm of per capita GDP (*lngdpper*) and logarithm of urban land area (*lnarea*) were controlled during 2012 to 2019. As the characteristics of the listed company itself may affect the risk appetite of the company, the relevant characteristics of the listed company are controlled. According to the general practices in the existing literatures, the company size of the listed company (*lnasset*) is controlled in the logarithmic form of total assets, and the survival period of the listed company is controlled in the logarithmic form (*lnage*).The company controls return on assets (*ROA*), Tobin’s Q value (*TBQ*), total assets growth rate (*TotalAssetGR*), net profit growth rate (*NetProfitGR*), revenue growth rate (*RevenueGR*), financial leverage ratio (*FinancialLR*) and the shareholding ratio of largest shareholders (*TOP*1));Personal characteristics of CEOs may also affect CEOs’ risk appetite. For example, the influence of education level may reduce risk appetite. Therefore, the logarithm of CEO’s age (*lnCEOage*), gender of CEO (*Gender*) and education level (*Schooling*) are controlled. Among them, the level of education is divided into 7 files according to the level of education from low to high: "uneducated": 0, primary school education, private school: 1, junior high school, technical school: 2, high school, vocational high state, secondary school, junior college (Adult), other: 3, junior college (unified), undergraduate (Adult): 4, undergraduate (unified): 5, postgraduate or above:6. The processing of “other” as 3 is based on the mean position, and the amount of “other” data is small (single digit), which does not greatly affect the overall data regression results; Finally, state-owned enterprises (*Gov*) were selected as the classification criteria for heterogeneity analysis. And the names and definitions of the above variables are shown in Table A.1 of S1 Appendix.

#### 2.2.4 Model specification

In order to examine the influence of literary inquisition on the risk appetite of CEOs in modern enterprises, this paper sets the following regression model:

$$Y_{i}=\alpha_{0}+\alpha_{1}{Literary}_{i}+\gamma X_{i}+\lambda_{p}+\varepsilon_{i}$$ (1)

Where *i* represents enterprise *i*, *Y*_*i*_ and Literary_*i*_ are respectively the risk preference of the CEO of enterprise i and whether the prefecture-level city where he was born has ever had a literary inquisition, *X*_*i*_ represents a set of control variables related firms. *λ*_*p*_ is province fixed effect and *ε*_*i*_ is the error term. If *α*_1_ is significantly less than 0, that is, literary investigation is negatively correlated with CEO risk preference, which is consistent with the prediction of the hypothesis; otherwise, the hypothesis is not valid.

In model (1), based on the mixed cross-sectional regression of CEO samples, Literary_*i*_’s estimated coefficient only reflects the basic influence of literary inquisition on risk appetite, ignoring the change of influence at different time stages, and cannot reflect the stability of whether the change of risk appetite over time is affected by literary inquisition during the performance of the same CEO. Therefore, by controlling the time effect and regional characteristic effect, the random effect model is adopted to further investigate the stability of literary inquisition influence. The model is set as follows:

$$Y_{i,t}=\beta_{0}+\beta_{1}{Literary}_{i}+\gamma X_{i,t}+\lambda_{p}+\lambda_{t}+\varepsilon_{i,t}$$ (2)

Where *Y*_*i*,*t*_ is the risk preference of the CEO of enterprise *i* in year t, *λ*_*p*_ and *λ*_*t*_ respectively represent province and year fixed effects and *ε*_*i*,*t*_ is the error term. Model (2) is based on the change in risk appetite of a single CEO over a period of different years, and examines the influence of literary inquisitorial. If the sign and significance of *β*_1_ are consistent with that of *α*_1_, it indicates that the results of model (1) are still valid after controlling for differences in time effects.

#### 2.2.5 Descriptive statistics

Table 1 provides a statistical description of the above variables. *LiteraryIB* is the literary inquisition characteristic quantity of CEO birthplace, the mean value is 0.42, and the standard deviation is about 0.49, indicating that the influence of literary inquisition index on CEO birthplace is relatively wide. *RiskAVar* is the risk preference of CEOs of enterprises, whose mean value is 0.0325 and standard deviation is 0.0869, indicating that CEOs of listed companies still have obvious differences in risk preference, which can be seen from the maximum and minimum values.

**Table 1:** Descriptive statistical results.

Variable name	Observed	Mean	Standard deviation	Minimum	Maximum	Median
**RiskAVar**	2,737	.03249545	.046815085	.00026823	.42597037	.0175273
**RiskARan**	2,737	.06127434	.086872023	.00050420	.81810430	.0333569
**LiteraryIB**	2,737	.42236025	.493935288	0	1	0
**LiteraryIN**	2,737	.42345634	.494106333	0	1	0
**LiteraryICB**	2,737	.1962002	.3971943	0	1	0
**LiteraryICN**	2,737	.2038729	.4029492	0	1	0
**lnCEOage**	2,737	3.916143	.1327795	3.332205	4.304065	3.931826
**Gender**	2,737	.9440994	.2297717	0	1	1
**Schooling**	2,737	4.16551	1.501951	1	7	4
**lnasset**	2,737	22.43477	1.47415	17.01851	28.50853	22.21934
**lnage**	2,737	2.30254	.6831201	.6931472	3.332205	2.484907
**Roa**	2,737	.0336275	.2210164	-6.776046	8.441391	.031996
**TBQ**	2,737	2.056266	2.14361	0	69.24426	1.574571
**TotalAssetGR**	2,737	.1585389	.4073218	-.870067	10.02865	.09056
**NetProfitGR**	2,737	-.6811874	74.27409	-3178.962	1829.021	0
**RevenueGR**	2,737	.8143133	15.36168	-3.256276	741.4094	.13303
**FinancialLR**	2,737	1.542534	3.781129	-14.32368	113.2739	1.085252
**Top1**	2,737	35.29566	15.03964	2.197	85.23	33.55
**lngdpper**	2,737	11.32556	.5853588	9.2193	15.6752	11.40649
**lnarea**	2,737	9.078544	.801151	7.090077	11.37231	9.191769
**lnpop**	2,737	6.412259	.643743	3.044523	8.132707	6.48219
**Gov**	2,737	.4139569	.4926309	0	1	0

## 3 Empirical result

### 3.1 Baseline finding

Table 2 shows the empirical results of the influence of risk appetite on literary inquisition. Columns (1)—(4) are the regression results of the estimation model (1). Univariate regression is performed for *RiskAVar*_*i*_ and *LiteraryIB* in column (1), and the regression coefficient is -0.00919. Using robust standard error, the results are significant at the 1% level. In column (5), the random effects model was used to conduct univariate regression for *RiskAVar*_*i*,*t*_ and *LiteraryIB*. The regression coefficient was -0.00985, and the result was still significant at the 1% level, indicating that under different regression models, the influence of literary investigation had a significant negative impact on CEO’s risk appetite. In column (2) to (4), the mixed cross-sectional regression results were successively added with CEO characteristic control variables, company control variables and regional control variables, and the regression coefficients were -0.0917, -0.0825 and -0.0870, respectively. The regression results were significant and stable. Considering the influence of time effect, (6)—(8) are listed as the regression results after successively increasing CEO characteristic control variables, company control variables and regional control variables, with regression coefficients of -0.00978, -0.00873and -0.00875, respectively, and the regression results are still significantly stable. This is perfectly consistent with hypothesis 1.

**Table 2:** Basic regression results.

Variable	RiskAVar_i_				RiskAVar_i,t_			
	(1)	(2)	(3)	(4)	(5)	(6)	(7)	(8)
**LiteraryIB**	-.00919*** (.00173)	-.00917*** (.00171)	-.00825*** (.00164)	-.00870*** (.00167)	-.00985*** (.00200)	-.00978*** (.01975)	-.00873*** (.00198)	-.00875*** (.00196)
**lnCEOage**		-.01449 (.00918)	-.01053 (.00970)	-.01185 (.00975)		-.01928** (.00959)	-.01304 (.00971)	-.01303 (.00963)
**Gender**		-.00268 (.00373)	-.00259 (.00348)	-.00216 (.00343)		.00177 (.00364)	.00165 (.00343)	.00168 (.00339)
**Schooling**		.00129** (.00064)	.00117* (.00063)	.00108 (.00066)		-.00107 (.00075)	-.00106 (.00074)	-.00107 (.00077)
**lnasset**			-.00434*** (.00078)	-.00450*** (.00077)			-.00487*** (.00071)	-.00487*** (.00072)
**lnage**			.00365** (.00150)	.00391*** (.00147)			.00223 (.00138)	.00225 (.00143)
**Roa**			-.02630 (.02009)	-.02643 (.02012)			-.02627 (.02069)	-.02627 (.02069)
**TBQ**			.00115*** (.00031)	.00109*** (.00031)			.00135*** (.00034)	.00134*** (.00033)
**TotalAssetGR**			-.00396 (.00364)	-.00395 (.00359)			-.00326 (.00324)	-.00327 (.00325)
**NetProfitGR**			-.00001 (.00001)	-.00001 (.00001)			-.00001 (.00001)	-.00001 (.00001)
**RevenueGR**			-.00004 (.00003)	-.00004* (.00002)			-00002 (.00003)	-.00002 (.00003)
**FinancialLR**			-.00032 (.00019)	-.00030 (.00019)			-.00020 (.00016)	-.000020 (.00017)
**Top1**			-.00030*** (.00006)	-.00029*** (.00006)			-.00024*** (.00007)	-.00024*** (.00007)
**lnarea**				.00065 (.00164)				.00017 (.00152)
**lngdpper**				.00505** (.00232)				.00042 (.00232)
**lnpop**				-.00148 (.00146)				-.00070 (.00134)
**constant**	.04222*** (.00073)	.09672*** (.03665)	.18081*** (.03515)	.13506*** (.03827)	.02501*** (.00285)	.09785*** (.03709)	.18632 *** (.03470)	.18768*** (.04587)
**Obs**	2737	2737	2737	2737	2737	2737	2737	2737
**R^2^**	0.0266	0.0303	0.0924	0.0960	0.070	0.0747	0.1381	0.1382

Note: Robust standard errors clustered at the city level are reported in parentheses.

*, ** and *** represent significant at the level of 10%, 5% and 1% respectively.

Through the joint results of the mixed cross-sectional regression and random effects model, it is inferred that the influence of the explosive site of literary inquisition on the informal system in this region has not weakened with the passage of time, and it is preliminary confirmed that this influence has formed the cultural “brand” of the population in this region and has been affected till now. In order to further confirm the reliability of this effect, In order to further confirm the reliability of this effect, it is necessary to conduct a robustness test to rule out the chance of this empirical result.

### 3.2 Robustness test

#### 3.2.1 Substitute the explained variable

The core explained variable is calculated using the variance method (*RiskAVar*) in the calculation process, and there is a uniformity of calculation methods. Risk preference (*RiskARan*) is recalculated using the range method again and the original explained variable is replaced. Mixed cross-sectional regression is used in columns (1)—(4) of Table 3. Similarly, columns (5)—(8) used a random effects model for regression. From the regression results, although the regression coefficients of the two different calculation methods have significantly changed, the significance of the regression results has not changed, and all the regression results are still negatively significant at the level of 1%. This result confirms that the results of different calculation methods of risk appetite do not affect the robustness of the study relationship.

**Table 3:** Alternative explained variables (range method).

Variable	RiskARan_i_				RiskARan_i,t_			
	(1)	(2)	(3)	(4)	(5)	(6)	(7)	(8)
**LiteraryIB**	-.01687*** (.00320)	-.01686*** (.00318)	-.01512*** (.00304)	-.01594*** (.00310)	-.01811*** (.00366)	-.01798*** (.00363)	-.01604*** (.00363)	-.01603*** (.00358)
**CEOCrcter**	NO	YES	YES	YES	NO	YES	YES	YES
**CompCtrl**	NO	NO	YES	YES	NO	NO	YES	YES
**RegionCtrl**	NO	NO	NO	YES	NO	NO	NO	YES
**Constant**	.07977*** (.00136)	.17574*** (.06669)	.33391*** (.06418)	.25118** (.06989)	.04751*** (.00531)	.17793*** (.06769)	.34441*** (.06338)	.35012*** (.08381)
**Obs**	2737	2737	2737	2737	2737	2737	2737	2737
**R^2^**	0.0266	0.0301	0.0926	0.0960	0.0711	0.0753	0.1389	0.1391

Note: Robust standard errors clustered at the city level are reported in parentheses.

*, ** and *** represent significant at the level of 10%, 5% and 1% respectively.

In addition to testing the robustness of the choice of risk preference method, the next test also switches the data time interval and conducts a robustness test. In the process of data selection, this paper uses the data results of listed companies for three consecutive years to calculate the risk appetite for the current period. In the results of basic regression, risk preference results are calculated using the method of advancing two periods. If risk volatility is recalculated with a one-period lag, will the regression results still be significant? After calculation and classification regression, the regression results in Table 4 are obtained.

**Table 4:** Substitute explained variables (difference time calculation).

Variable	RiskAVar_i_ (t-1_t+1)				RiskAVar_i,t_ (t-1_t+1)			
	(1)	(2)	(3)	(4)	(5)	(6)	(7)	(8)
**LiteraryIB**	-.00450* (.00237)	-.00462** (.00231)	-.00423** (.00190)	-.00451** (.00187)	-.00486** (.00230)	-.00489** (.00223)	-.00431** (.00179)	-.00435** (.00180)
**CEOCrcter**	NO	YES	YES	YES	NO	YES	YES	YES
**CompCtrl**	NO	NO	YES	YES	NO	NO	YES	YES
**RegionCtrl**	NO	NO	NO	YES	NO	NO	NO	YES
**constant**	.02325*** (.00169)	.03903 (.02951)	.12872 (.02755)	.07995* (.04124)	.01796*** (.00196)	.05112*** (.02978)	.14482 (.02734)	.16191*** (.04965)
**Obs**	2737	2737	2737	2737	2737	2737	2737	2737
**R^2^**	0.0251	0.0275	0.1067	0.11	0.0676	0.0699	0.1539	0.1545

Note: Robust standard errors clustered at the city level are reported in parentheses.

*, ** and *** represent significant at the level of 10%, 5% and 1% respectively.

Columns 1–4 show the robustness of the calculation results of risk appetite with a delay of one period by using a mixed cross-sectional regression method, which can be seen to be significant at the level of 5%. In the same way, columns 5–8 show the robustness of the calculation results of risk preference with a delay of one period by using random effects regression, and the results are also significant above 5%, basically passing the robustness test.

#### 3.2.2 Alternative explanatory variable

If literary inquisition has an impact on CEOs’ risk appetite, not only the place of birth will have an impact on their growth, but also the place of birth will change their risk appetite through family cultural influence and intergenerational influence. Substitute explanatory variables are obtained by matching the place of literary inquisition of CEOs’ native place (*LiteraryIN*). This explanatory variable is used to regression the risk preference of CEOs of listed companies, and the regression results are shown in Table 5. In columns (1)—(4), mixed cross-sectional regression is adopted, and it can be seen that the regression results are significantly negative at the level of 1%, which is consistent with the basic regression results. In columns (5)—(8), the random effects model was used for regression, and the same results maintained negative significance at the level of 1%, further proving the robustness of the inference.

**Table 5:** Alternative main explanatory variables (matched by place of origin).

Variable	RiskAVar_i_				RiskAVar_i,t_			
	(1)	(2)	(3)	(4)	(5)	(6)	(7)	(8)
**LiteraryIN**	-.00930*** (.00174)	-.00935*** (.00174)	-.00872*** (.00169)	-.00907*** (.00171)	-.00991*** (.00201)	-.00993*** (.00203)	-.00924*** (.00211)	-.00924*** (.00208)
**CEOCrcter**	NO	YES	YES	YES	NO	YES	YES	YES
**CompCtrl**	NO	NO	YES	YES	NO	NO	YES	YES
**RegionCtrl**	NO	NO	NO	YES	NO	NO	NO	YES
**constant**	.00423*** (.00074)	.09922*** (.03699)	.18445*** (.03559)	.13982*** (.03872)	.02502*** (.00290)	.10053** (.03798)	.19022*** (.03585)	.19263*** (.04703)
**Obs**	2737	2737	2737	2737	2737	2737	2737	2737
**R^2^**	0.0268	0.0306	0.0933	0.967	0.0705	0.0750	0.1391	0.1392

Note: Robust standard errors clustered at the city level are reported in parentheses.

*, ** and *** represent significant at the level of 10%, 5% and 1% respectively.

Further, in order to more accurately observe the influence of literary investigation on the risk appetite of CEOs of listed companies, The next test uses county data for matching to obtain explanatory variables (*LiteraryICB*), and also used birthplace and native place to divide and compare. Although only some data in the final matching results were matched, it was enough to test the robustness. Columns (1)—(4) shown in Table 6 are regression results based on the CEO birthplace matched by counties as explanatory variables. From the result, when the full control variables are added, column (4) shows negative significance at 1% level; Columns (5)—(8) were matched by the native place of the CEO of the listed company, and a negative significant result at the 1% level was also obtained after the addition of control variables. Using precise geographical methods, the results show that the influence of literary prison on the risk appetite of modern corporate CEOs is still significant and robust.

**Table 6:** Alternative main explanatory variables (county matching).

Variable	RiskAVar_i_							
	Birthplace				Nativeplace			
	(1)	(2)	(3)	(4)	(5)	(6)	(7)	(8)
**LiteraryIC**	-.00375** (.00190)	-.00392** (.00190)	-.00451** (.00195)	-.00536*** (.00203)	-.00437** (.00191)	-.00456** (.00194)	-.00534*** (.00202)	-.00610*** (.00208)
**CEOCrcter**	NO	YES	YES	YES	NO	YES	YES	YES
**CompCtrl**	NO	NO	YES	YES	NO	NO	YES	YES
**RegionCtrl**	NO	NO	NO	YES	NO	NO	NO	YES
**constant**	.03901*** (.00035)	.09949*** (.03735)	.18567*** (.03624)	.14550*** (.03896)	.03912*** (.00035)	.10061*** (.03768)	.18753*** (.03670)	.14752*** (.03921)
**Obs**	2737	2737	2737	2737	2737	2737	2737	2737
**R^2^**	0.0183	0.0221	0.0865	0.897	0.0187	0.0225	0.0871	0.0904

Note: Robust standard errors clustered at the city level are reported in parentheses.

*, ** and *** represent significant at the level of 10%, 5% and 1% respectively.

## 4 Heterogeneity analysis

### 4.1 Research on heterogeneity of firm nature

In all listed companies in China, from the nature of enterprise holding, it can be roughly divided into two categories: one is state-owned enterprise holding company, the other is non-state-owned enterprise holding company. From the perspective of CEO’s executive duties, non-state-owned enterprise holding companies pay more attention to corporate benefits, profits and shareholders’ rights and interests, so risk management tends to be risk aversion, which will be further strengthened among CEOs who have been influenced by literary inquisition culture. While state-owned holding enterprises pay more attention to non-profit business objectives such as social benefits and employee welfare. Therefore, from the perspective of heterogeneity research, non-state-owned enterprises should have more significant risk aversion characteristics.

In this part, 2737 observations were divided into 1133 observations of state-owned enterprises and 1604 observations of non-state-owned enterprises through the firm nature variable (*Gov*), and then carried out classification regression. In the same way, two basic regression methods were used in the regression process, and empirical results in Table 7 were obtained. From the empirical results, the first four columns are regression results of non-state-owned enterprises. In the results of mixed cross-sectional regression, risk appetite (*RiskAVar*) and literary inquisition index (*LiteraryIB*) show significant negative correlation regardless of variable regression or increased control variable regression, and the same result also appears in the random effects regression results. However, after classifying SOEs, no matter what model is used, the regression results are not significant, that is, the brand of literary investigation does not effectively inhibit the risk appetite of CEOs of SOEs. The above results can fully confirm our second basic hypothesis: the negative impact of the literary trial incident on CEO risk appetite is significantly greater in private enterprises than in state-owned enterprises.

**Table 7:** Research on heterogeneity of enterprise nature.

Variable	Private enterprise				State-owned enterprise			
	RiskAVar_i_	RiskAVar_i,t_	RiskAVar_i_	RiskAVar_i,t_
**LiteraryIB**	-.01324*** (.00217)	-.01083*** (.00226)	-.01418*** (.00220)	-.01092*** (.00219)	-.00282 (.00202)	-.00277 (.00192)	-.00300 (.00204)	-.00271 (.00189)
**CEOCrcter**	YES	YES	YES	YES	YES	YES	YES	YES
**CompCtrl**	NO	YES	NO	YES	NO	YES	NO	YES
**RegionCtrl**	NO	YES	NO	YES	NO	YES	NO	YES
**constant**	.04323 (.04702)	.02617 (.04942)	.04565 (.04371)	.11320** (.04598)	.17420*** (.04175)	.11103** (.05072)	.17995*** (.04154)	.14729*** (.05187)
**Obs**	1,604	1,604	1,604	1,604	1,133	1,133	1,133	1,133
**R^2^**	0.0349	0.2046	0.1006	0.2557	0.0662	0.1674	0.0783	0.1797

Note: Robust standard errors clustered at the city level are reported in parentheses.

*, ** and *** represent significant at the level of 10%, 5% and 1% respectively.

### 4.2 Study on the heterogeneity of marketization level

To some extent, the marketization level of listed companies determines the standardization of market operation and the risk management ability of listed companies when dealing with risks. Generally, it is believed that the higher the level of marketization, the stronger the enterprise risk management ability, which indirectly affects the result of risk appetite. In order to further investigate the interaction between the formal system (marketization index) and the informal system (literary inquisition), the samples were grouped according to the degree of marketization (*Market*) and then regression analysis was carried out. The results are shown in Table 8. First of all, it can be seen from Table 8 that the significant negative impact of literary inquisition variable on enterprise risk preference exists only in regions with relatively high degree of marketization. In the regions with relatively low degree of marketization, the negative influence of literary inquisition on enterprise risk preference is not significant. It can be seen that there is a certain complementary relationship between literary inquisition (informal system) and market index (formal system).Secondly, it shows that the function of informal institutions depends on the construction of formal institutions, and market friction and failure cannot be solved by informal institutions such as the literary inquisition incident, while the construction of formal institutions with infrastructure significance cannot be ignored.

**Table 8:** Regression analysis based on the marketization index median grouping.

Variable	High market area enterprises				Enterprises in low marketization areas			
	RiskAVar_i_		RiskAVar_i,t_		RiskAVar_i_		RiskAVar_i,t_	
**LiteraryIB**	-.00898*** (.00179)	-.00861*** (.00177)	-.00971*** (.00189)	-.00878*** (.00182)	-.01315 (.00792)	-.00664 (.00662)	-.01125 (.00834)	-.00723 (.00704)
**CEOCrcter**	YES	YES	YES	YES	YES	YES	YES	YES
**CompCtrl**	NO	YES	NO	YES	NO	YES	NO	YES
**RegionCtrl**	NO	YES	NO	YES	NO	YES	NO	YES
**constant**	.08241** (.03885)	.12567*** (.03872)	.08562** (.03777)	.17693*** (.03879)	.25530** (.12746)	.19427 (.14052)	.24039** (.12002)	.20489 (.14027)
**Obs**	2,530	2,530	2,530	2,530	207	207	207	207
**R^2^**	0.0321	0.0937	0.0766	0.1354	0.1112	0.5130	0.2089	0.5444

Note: Robust standard errors clustered at the city level are reported in parentheses.

*, ** and *** represent significant at the level of 10%, 5% and 1% respectively.

## 5 Competitive hypothesis

### 5.1 The influence of Confucian culture on risk appetite

In areas where Confucian culture is flourishing, modern entrepreneurs are conservative under the influence of Confucian culture [3].As an important informal system, Confucian culture has attracted wide attention from scholars at home and abroad. In this paper, by collecting the data of 25,229 catalogs of Jinshi in the Qing Dynasty, the logarithm of the number of Jinshi in corresponding regions was obtained according to their regional distribution (*Lnjinshi*), and the density of Jinshi in each region (*JinshiDen*, Jinshi / Per 10,000 people) was calculated using the regional population data of 2018.The regression is carried out as proxy variables of Confucian culture respectively, and the regression results are shown in Table 9:

**Table 9:** Literary inquisition and Confucian culture.

Variable	Number of jinshi				Jinshi density			
	RiskAVar_i_		RiskAVar_i,t_		RiskAVar_i_		RiskAVar_i,t_	
**LiteraryIB**	-.00916*** (.00171)	-.00869*** (.00168)	-.00976*** (.00196)	-.00874*** (.00195)	-.00912*** (.00173)	-.00866*** (.00169)	-.00973*** (.00199)	-.00872*** (.00197)
**lnjinshi**	-.00037 (.00080)	-.00044 (.00075)	-.00032 (.00063)	-.00033 (.00062)				
**JinshiDen**					-.00385* (.00211)	-.00367* (.00211)	-.00317** (.00140)	-.00288* (.00151)
**CEOCrcter**	YES	YES	YES	YES	YES	YES	YES	YES
**CompCtrl**	NO	YES	NO	YES	NO	YES	NO	YES
**RegionCtrl**	NO	YES	NO	YES	NO	YES	NO	YES
**constant**	.09889*** (.03903)	.13721*** (.03983)	.09966*** (.03850)	.18917*** (.04692)	.09826*** (.03656)	.13612*** (.03830)	.09900*** (.03679)	.18825*** (.04574)
**Obs**	2737	2737	2737	2737	2737	2737	2737	2737
**R^2^**	0.0304	0.961	0.0748	0.1383	0.0309	0.0965	0.0751	0.1386

Note: Robust standard errors clustered at the city level are reported in parentheses.

*, ** and *** represent significant at the level of 10%, 5% and 1% respectively.

The results show that the number of Jinshi does not affect enterprise risk preference, and the density of Jinshi inhibits enterprise risk preference at the level of 10%.This confirms the inhibitory effect of Confucian culture on risk preference. However, with the addition of the variable of the density of scholars, the influence of literary inquisition on risk preference remains significant at the level of 1%, indicating that the influence of Confucian culture and literary inquisition on the risk preference of CEOs of modern enterprises is two different paths, and there is no substitute relationship between them. The influence of Confucian culture on risk appetite will not be discussed in depth here.

### 5.2 The influence of modern business credit environment

CEOs’ risk bias is also closely related to the business credit environment, and the business credit level of the location of an enterprise is the embodiment of the relationship between the overall corporate execution and social credit in a region. In this paper, the China City Commercial Credit Environment Index (*CEI*) is selected to match the location of enterprises, and incorporated the modern business credit environment index into the systematic regression. Through multiple classification regression, the substitution relationship between modern business credit environment and literary investigation incident was basically excluded. The regression results are shown in Table 10:

**Table 10:** Regression results of CEI.

Variable	RiskAVar_i_				RiskAVar_i,t_			
**LiteraryIC**	-.00919*** (.00173)	-.00917*** (.00171)	-.00825*** (.00164)	-.00870*** (.00167)	-.00985*** (.00180)	-.00977*** (.00179)	-.00874*** (.00171)	-.00875*** (.00171)
**CEI**	.00001 (.00023)	.00002 (.00023)	.00013 (.00023)	.00012 (.00023)	.00001 (.00021)	.00001 (.00020)	.00010 (.00020)	.00010 (.00020)
**CEOCrcter**	NO	YES	YES	YES	NO	YES	YES	YES
**CompCtrl**	NO	NO	YES	YES	NO	NO	YES	YES
**RegionCtrl**	NO	NO	NO	YES	NO	NO	NO	YES
**constant**	.04324*** (.01957)	.09809** (.04539)	.19215*** (.04344)	.14562*** (.04363)	.02399*** (.01828)	.09729*** (.04353)	.19462** (.04165)	.19621*** (.04306)
**obs**	2737	2737	2737	2737	2737	2737	2737	2737
**R^2^**	0.0266	0.0303	0.0925	0.0960	0.0704	0.0747	0.1381	0.1383

Note: Robust standard errors clustered at the city level are reported in parentheses.

*, ** and *** represent significant at the level of 10%, 5% and 1% respectively.

## 6 Mechanism study

The reason for the above impact, this paper believes that the main reason is that the precipitation of literary inquisition caused a certain reduction in the overall social capital of the region. The study found that charity, public welfare institutions and social trust in the literary inquisition area had a corresponding weakening effect. Among them, the influence of social trust is the key factor that has an important impact on risk appetite. When the social trust decreases, people’s awareness of risk aversion will increase, risk appetite will be significantly reduced; On the contrary, when social trust increases and social capital accumulates rapidly, risk appetite will increase significantly. In order to test the mechanism, this paper uses the survey data from the 2015 Chinese General Social Survey (CGSS) to match the prefecture-level cities where literary inquiry is located and uses OLS basic regression to test. It should be noted that some cities could not obtain complete matching results because they did not participate in the survey, but the empirical test of the partial matching data still shows that literary inquiry has a negative impact on trust in the broad and narrow sense of the region. Specific research methods are as follows:

### 6.1 Explained variable

The trust degree of each region in the questionnaire is studied as the core explained variable. With reference to existing literatures, trust is divided into two categories. The first category is broad social trust (*GenSocialTr*). The main survey data are taken from question a33 in the questionnaire: “Generally speaking, do you agree or disagree with the opinion that most people in the society can be trusted”. According to the data processing of the survey results, the answer “strongly agree” is equal to 5, “relatively agree” is equal to 4, “cannot agree to disagree” is equal to 3, “relatively disagree” is equal to 2, “strongly disagree” is equal to 1, that is, high is divided into trust, low is divided into distrust, a total of 10927 valid data are obtained, which is used to reflect the broad social trust.

The other category is the narrow sense of social trust (*NarSocialTr*), which aims at the trust degree of specific objects. A total of 13 types of objects are selected for investigation in this questionnaire, involving questions b1001 to b1013 in the questionnaire: “Trust degree in general social interactions/contacts that do not directly involve pecuniary interests -”. After the same data processing for the answers to the above questions, 10,927 observed values of each type of data were obtained. Similarly, the high scores had higher trust and the low scores had lower trust.

### 6.2 Explanatory variable

Whether there had ever been a literary inquisition in the city (*LiteraryIB*) was taken as the core explanatory variable, and its influence on trust was paid attention to through linear OLS regression.

### 6.3 Control variables

Control variables choose personal characteristics variables and regional control variables two categories. Among the personal characteristic variables, the logarithm of the respondents’ age (*lnage*), gender (*Gender*), educational background (*Schooling*), religious belief (*Religion*), political status (*PoliticSt*) and other data were selected. The education level of the visitor is processed by data, and the education level is divided into 7 files from low to high: ‘uneducated’: 0, primary education, private school: 1, junior high school, technical school: 2, high school, vocational high state, secondary school, junior college (adult), other: 3, junior college (unified), undergraduate (adult): 4, undergraduate (unified): 5, postgraduate or above: 6. The processing of “other” as 3 is based on the mean position, and the amount of “other” data is small (single digit), which does not greatly affect the overall data regression results; Gender is treated as dummy variable, male is 1, female is 0;Religious belief is treated as dummy variable, with religious belief as 1 and no religious belief as 0;The political status is treated as categorical variable, with the mass and inability to answer as 0, the Communist Youth League members as 1, the democratic parties as 2, and the Communist Party members as 3; Age is treated logarithmically. In addition, the logarithm of the area of prefecture-level cities (*lnarea*), logarithm of per capita GDP (*lngdpper*) and logarithm of population (*lnpop*) were used to control the regional characteristics of respondents. And the names and definitions of all the variables used in this paper are displayed in Table A.1 of S1 Appendix.

Table 11 shows the results of OLS based regression based on sample data. Column (1)—(3) is the regression result of generalized trust. From the univariate regression result, literary inquisition has a significant negative impact on social generalized trust at 5% level, and after controlling by adding individual characteristic effect and regional effect, the result still shows a relatively stable and significant result. Column (4) — (6) show the regression results of relative trust (*NarSocialTr*_*Rel*) in narrow sense of social trust, which are univariate regression, controlling for individual characteristics and controlling for regional effect respectively. The results show that the region where the prison has occurred has a very significant negative effect on relative trust (significant at 1% level). Columns (7)—(9) are the regression results of neighbor trust (*NarSocialTr*_*Nei*), which are also the results from univariate regression to incremental control variable regression. From the results, the negative trend is also significant at the 1% level. Except for the two narrow sense social trust with significant negative correlation, the empirical results of other social relationships are not significant. This paper gives an example of one kind of social trust in the narrow sense, and takes the classmate relationship (*NarSocialTr*_*Mat*) as an example for regression. The empirical results in columns (9)—(12) reflect the influence relationship of social trust in this narrow sense. Neither univariate regression nor the regression results with additional control variables are significant, or it didn’t have the significant effect that was assumed.

**Table 11:** Empirical results of social trust (CGSS2015 survey data).

Variable	GenSocialTr			NarSocialTr_Rel			NarSocialTr_Nei			NarSocialTr_Mat		
	(1)	(2)	(3)	(4)	(5)	(6)	(7)	(8)	(9)	(10)	(11)	(12)
**LiteraryIB**	-.04192** (.02042)	-.05372*** (.02044)	-.04811** (.02057)	-.06369*** (.01808)	-.06599*** (.01825)	-.06452*** (.018279)	-.09157*** (.02029)	-.07889*** (.020420)	-.07416*** (.02051)	.02976 (.02557)	-.04577* (.02428)	-.04187* (.02437)
**lnage**		.37026*** (.02936)	.37334*** (.02939)		-.05099** (.024641)	-.04550* (.02468)		.01908 (.02825)	.03157 (.02825)		-.23440*** (.03137)	-.23153*** (.03148)
**Gender**		.04112** (.01862)	.03977** (.01862)		-.02394 (.01622)	-.02528 (.01621)		.01912 (.01810)	.01522 (.01808)		.14002*** (.02169)	.13928*** (.02169)
**Religion**		.00766 (.03093)	.01058 (.03095)		.01915 (.02633)	.01484 (.02636)		-.00668 (.02976)	-.00882 (.02977)		-.07159* (.03799)	-.07351* (.03802)
**Schooling**		.02205*** (.00820)	.02429*** (.00830)		.00388 (.00698)	.00689 (.00705)		-.04843*** (.00786)	-.04108*** (.00793)		.25070*** (.00941)	.25246*** (.00949)
**PoliticSt**		.03832*** (.01050)	.03848*** (.01051)		.03704*** (.00926)	.03596*** (.00923)		.02151 (.01051)	.02024 (.09463)		.03764*** (.01147)	.03708*** (.01147)
**lnpop**			.03770 (.02533)			.08024*** (.02292)			.16798*** (.02443)			.04356 (.03210)
**lnarea**			.02381 (.01985)			-.06364*** (.01753)			-.08488*** (.01968)			-.01634 (.02460)
**lngdpper**			.09024*** (.02863)			.06236** (.02704)			.11079*** (.02783)			.09304** (.03877)
**constant**	3.4831*** (.01081)	1.9799*** (.12270)	.58195 (.41473)	4.2468*** (.00917)	4.4317*** (.10219)	3.8631*** (.39373)	3.9085*** (.01026)	3.9144*** (.11776)	2.4884*** (.40116)	3.3611*** (.01359)	3.7013*** (.12995)	2.6053*** (.56398)
**obs**	10,927	10,927	10,927	10,927	10,927	10,927	10,927	10,927	10,927	10,927	10,927	10,927
**R** ^**2**^	0.0004	0.0208	0.0218	0.0012	0.0038	0.0068	0.0020	0.0073	0.0121	0.0001	0.1266	0.1274

Note

*, ** and *** represent significant at the level of 10%, 5% and 1% respectively.

The broad sense of social trust has a significant negative impact on the influence of literary inquisition, which indicates that hypothesis 3 of this paper: Literary inquisition incident reduces the risk appetite of the people in a region by reducing the social trust, which is valid. However, in the narrow sense of social trust, there are more diversified differences, that is, some objects’ social trust will be reduced, while others’ social trust will not be significantly reduced. The explanation of the above regression results can be found from the detailed descriptions of various cases in the “Qing Dynasty Literary Inquisition Files”:

First, there are many cases in writing prison are directly launched by the current emperor, rather than by the stable social system to regulate the writing standards of individual characters, such as Hu Zhongzao “Jian Mosheng Shiqian” case, Xie Jishi book case, etc., this kind of literary inquisition has a strong subjectivity and randomness of the emperor, just as the folk so-called “accompanying the king like accompanying the tiger” sentiment. Therefore, social trust in a broad sense can be affected by the literary inquisition, while most social relations are not imprinted and precipitated by such a literary inquisition.

Second, although many cases in literary inquisition were initiated or approved by the emperor of the court, the cases often originated from the neighbors’ conflicts and mutual reports and eventually ferment into a large-scale literary inquisition; In addition, in the examination and investigation of literary inquisition, neighbors also become very important information from the conviction basis, which makes the relationship between neighbors become an important object to prevent risks. The saying “walls have ears” is a true portrayal, such as: Cheng Mingyin as Shou copywriter, Yu Tengjiao poetry case; Although “distant relatives are not as good as close neighbors”, once the incident, the nine ethnic groups may be affected, so the relationship between relatives is also an important basis for the conviction of literary inquisition involvement. Such as the case of Zhuang’s Ming History in the early Qing Dynasty, Zhuang’s family was almost slaughtered. Under such pressure, the only way to avoid disaster is to either declare first or publicly declare that the relationship has been severed.

Third, other social relationships were largely unaffected. For example, the classmate relationship is a kind of modern social relationship, which runs through the whole period of primary education to higher education. However, in the Qing Dynasty, the relationship between classmates only appeared in the period of primary education, and the relationship between classmates can be interpreted as classmates or classmates. In the cases of literary inquisition in the Qing Dynasty, classmates or classmates were not implicated, and there was no first report between classmates, which did not have a significant impact on the trust degree of the object.

Through the above analysis, it can be more confirmed that the important reason for the decline of social trust is the influence of literary inquisition, and these influences have been precipitated until now, affecting a series of modern social elements including the risk preference of corporate CEOs, forming a cultural “brand”.

## 7 Conclusion and enlightenment

In recent years, more and more scholars have paid attention to the precipitation effect of history, and a large number of texts have reflected the potential impact of historical events on modern culture and economic behavior. This paper finds that the literary inquisition incident still has a significant negative impact on the risk appetite of modern enterprises after 100 years, and this conclusion remains unchanged after a robustness test and heterogeneity study. With the help of the survey data of 2015 Chinese General Social Survey (CGSS2015), further in-depth discussion is carried out. The mechanism study proves that the influence of literary inquiries is derived from the influence on social trust, which ultimately makes the residents of this area more inclined to conservative risk preference.

The research conclusions of this paper have the following implications:

First, culture has a long-term impact on the behavior of entrepreneurs. The research conclusion of this paper confirms that the influence of history and culture on the risk preference of CEOs of modern enterprises exists and is significant. How to use modern policy means to improve the difference in social capital between regions also provides a new reference.

Second, decision-makers’ risk appetite is influenced by multi-cultural and historical background. This study shows that the influence of literary inquiry can effectively reduce the risk appetite of CEOs, which is an important influence of the informal system. How to develop a good capital market is still an important topic for the construction of China’s future financial market, which is an important starting point for enterprise risk control. In addition to a more stringent market supervision mechanism, the impact of the informal system cannot be ignored, especially in the handling of major social events.

Third, there is still much to be explored in the interpretation of modern culture and institutions by historical events. Through the “precipitation” effect of literary inquisition, we can see that there are more and more important events in history, and the influence of these events may far exceed the influence of literary inquisition. The research in this paper also puts forward a feasible research idea for the study of other events.

## Supporting information

S1 Appendix Variable definitions. (DOCX)

S1 File (DTA)

S2 File (DTA)

S3 File (DTA)

S1 Data (XLSX)

S2 Data (XLSX)

S3 Data (XLSX)
