# Supplementary material for: ‘Forgetting’ or ‘Precipitation’: Literary inquisition in Qing Dynasty and modern enterprise risk preference
Source: PLoS One. 2024 Mar 22;19(3):e0300639. doi: 10.1371/journal.pone.0300639 (PMC10959367; doi:10.1371/journal.pone.0300639)
Supplement: S1 Appendix — (DOCX) [file pone.0300639.s001.docx]

# S1 Appendix. Variable definitions

Table A.1 The names and definitions of variables

| Notations | Names | Definitions |
| --- | --- | --- |
| Measures of dependent variables | |  |
| RiskAVar | Corporate risk-taking calculated by the deviation of Adj_ROA, | Use the volatility of corporate earnings as a proxy for risk-taking. ROA is the EBIT in the corresponding year divides the total assets at the end of the year. When calculating the volatility, the Adj_ROA is obtained by subtracting the annual industry average from the firm's ROA in each year, to mitigate the impact of the industry and the cycle (John et al., 2008; Yu et al., 2013). |
| RiskARan | Corporate risk-taking calculated by the range of Adj_ROA, | RiskARan is calculated by Range of Adj_ROA |
| GenSocialTr | General social trust | The main survey data is taken from the CGSS questionnaire question a33: "Generally speaking, do you agree or disagree with the majority of people in this society can be trusted?" According to the data processing of the survey results, the answer "strongly agree" is equal to 5, "relatively agree" is equal to 4, "can not agree to disagree" is equal to 3, "relatively disagree" is equal to 2, "strongly disagree" is equal to 1, that is, high is divided into trust, low is divided into distrust, a total of 10927 valid data are obtained, which is used to reflect the broad social trust. |
| NarSocialTr_Rel | Social trust in narrow sense, reflects trust between relatives | Using the survey results of questions b1001 to b1013 in the CGSS questionnaire: "Trust degree in general social interactions/contacts that do not directly involve pecuniary interests -", we conducted the same data processing for the answers to the above questions, and obtained 10,927 observed values of each type of data, with the same high score as higher trust degree and low score as lower trust degree. |
| NarSocialTr_Nei | Social trust in narrow sense, reflects the trust between neighbors |  |
| NarSocialTr_Mat | Social trust in narrow sense, reflects the trust between classmates |  |
| Measures of independent variables | |  |
| LiteraryIB | The dummy variables of literary inquisition in the birthplace of CEO in prefecture-level city | According to the “Qing Dynasty Literary Inquisition Files”, the birthplace of CEO of listed company is identified. The occurrence of literary inquisition is 1, and the absence of literary inquisition is 0, and the dummy variable is constructed |
| LiteraryIN | The dummy variables of literary inquisition in the birthplace of CEO in prefecture-level city | According to the data of literary inquisition in Qing Dynasty, the native place of CEO of listed company is identified. |
| LiteraryICB | The dummy variables of literary inquisition in the birthplace of CEO in County | Construct literary inquisition dummy variable at county level, birthplace. |
| LiteraryICN | The dummy variables of literary inquisition in the birthplace of CEO in County | Construct literary inquisition dummy variable at county level, native place. |
| Gov | Company ownership dummy variable | Nature of ownership of listed companies , collected and organized according to the Wind data database |
| Market | Prefecture-level Market development Index | Provincial Market development Index provided by Fan et al. (2018) |
| lnjinshi | The number of scholars in Qing Dynasty is a proxy variable of Confucian culture | The number of Jinshi in Qing Dynasty, using the "Qing Dynasty Jinshi Directory" collated prefecture-level city data, logarithm |
| JinshiDen | The density of scholars in Qing Dynasty, the proxy variable of Confucian culture | The density of Jinshi in Qing Dynasty was calculated per 10,000 people according to the population of prefecture-level cities in 2018, using the data of prefecture-level cities organized by the Directory of Jinshi in Qing Dynasty |
| CEI | China City Commercial Credit Environment Index | CEI is compiled by the CEI expert research group in China and can be used to evaluate the quality of a city's market credit environment |
| Measures of control variables | |  |
| lnCEOage | The logarithm of CEO age | Age of CEO of listed company, collected and organized according to the Wind data database |
| Gender | CEO gender of listed companies | The gender of CEO of listed companies, dummy variable, 1 is male, 0 is female, collected and organized according to the Wind data database |
| Schooling | The educational level of CEOs of listed companies | The level of education is divided into 7 grades according to the level of education from low to high: "uneducated" : 0, primary school education, private school: 1, junior high school, technical school: 2, high school, vocational high state, secondary school, junior college (Cheng), other: 3, junior college (unified), undergraduate (Cheng) : 4, undergraduate (unified) : 5, postgraduate above: 6collected and organized according to the Wind data database |
| lnasset | Assets of listed companies, logarithm | Assets of listed companies, logarithm, collected and organized according to the Wind data database |
| lnage | Duration of listed company, logarithm | Duration of listed company, logarithm, collected and organized according to the Wind data database |
| Roa | Return on assets of listed companies | Return on assets of listed companies, collected and organized according to the Wind data database |
| TBQ | Tobin Q value of listed companies | Tobin Q value of listed companies, collected and organized according to the Wind data database |
| TotalAssetGR | Growth rate of total assets of listed companies | Growth rate of total assets of listed companies, collected and organized according to the Wind data database |
| NetProfitGR | Net profit growth rate of listed companies | Net profit growth rate of listed companies, collected and organized according to the Wind data database |
| RevenueGR | Revenue growth rate of listed companies | Revenue growth rate of listed companies, collected and organized according to the Wind data database |
| FinancialLR | Financial leverage ratio of listed companies | Financial leverage ratio of listed companies, collected and organized according to the Wind data database |
| Top1 | The shareholding ratio of the largest shareholder of a listed company | The shareholding ratio of the largest shareholder of a listed company, collected and organized according to the Wind data database |
| lngdpper | Logarithm of GDP per capita | Per capita GDP of prefecture-level cities, collected and collated according to China Urban Statistical Yearbook |
| lnarea | Logarithm of area | Logarithm of area, collected and collated according to China Urban Statistical Yearbook |
| lnpop | Logarithm of regional population | Logarithm of regional population, collected and collated according to China Urban Statistical Yearbook |
